# Diagnosis of Paracardiac Castleman Disease by Dynamic Gadolinium-Enhanced First Pass Perfusion Magnetic Resonance Imaging

**DOI:** 10.4137/ccrep.s732

**Published:** 2008-10-01

**Authors:** Andrew Crean, Narinder Paul, Naeem Merchant, Lianne Singer, Yves Provost

**Keywords:** Castleman disease, first pass perfusion, gadolinium

## Abstract

Summary

Castleman disease is an uncommon disorder affecting the lymphatic system and is characterised by atypical lymphocyte proliferation. The usual clinical presentation is of a solitary mass lesion, frequently within the thorax. A number of different imaging findings have been reported on CT and MRI. We present a case of paracardiac Castleman disease where the diagnosis was suggested by dramatic enhancement of the tumour mass during a dynamic MR perfusion sequence. To our knowledge this is the first report of the use of a first pass bolus tracking technique in the diagnosis of Castleman disease.

## Case Report

The patient was a 35 year old woman with an extensive past medical history including pemphigus vulgaris. She had a previous diagnosis of Castleman disease confirmed by pathology following excision approximately 10 years earlier. She subsequently developed bronchiolitis obliterans and underwent bilateral lung transplantation. Subsequent CT follow up demonstrated a soft tissue mass in the mediastinum that remained stable for a number of years. Unfortunately in the decade after her lung transplant she again developed a clinical picture of bronchiolitis obliterans, this time attributed to chronic allograft rejection. Repeat double lung transplant was contemplated and she underwent coronary angiography as part of the pre-surgical work up. At catheterization a vascular mediastinal mass was discovered and MRI examination of the thorax was requested.

### MRI findings

**Figure 1:**
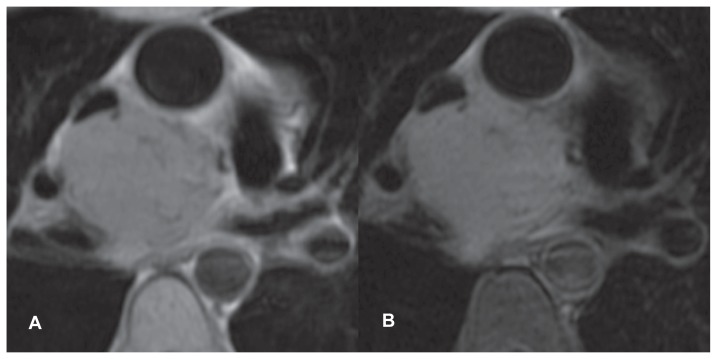
**A** (Double inversion recovery T1 pre-contrast), **B** (Double inversion recovery T2 fat-suppressed pre-contrast). The lesion is closely associated with multiple vascular structures in the mediastinum. Note the internal tubular areas of signal void (see text).

Pre-contrast imaging demonstrated a 5 × 3.8 × 5.2 cm solid mass in the middle mediastinum. This appeared homogenous and of slightly higher signal than skeletal muscle on T1 weighted images, with moderate signal intensity on fat-suppressed T2 weighted images (Figs. 1a/b). Subtle serpiginous areas of signal void were identified within the mass.

**Figure 2:**
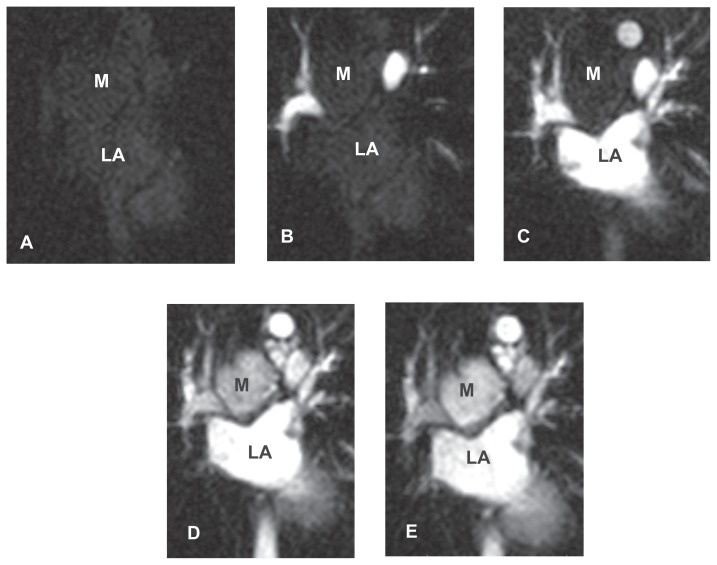
**A–E** Dynamic first pass perfusion sequence (FGRET) from time = 0 to time = 12 secs after gadolinium injection. The position of the nodal mass (M) is shown relative to the left atrium (LA) on this coronal acquisition.

A dynamic multi-slice first pass perfusion sequence was performed in the coronal plane (for imaging parameters see Table 1). Image acquisition was commenced immediately before an injection of 10 ml of gadolinium-DTPA and continued for approximately 80 heart beats. During this acquisition dramatic enhancement of the mass was seen to occur, with peak signal intensities only slightly less than those of the surrounding vascular structures and peak enhancement occurring 1–2 heartbeats after contrast appeared in the thoracic aorta (Figs. 2a–e images/movie).

**Figure 3:**
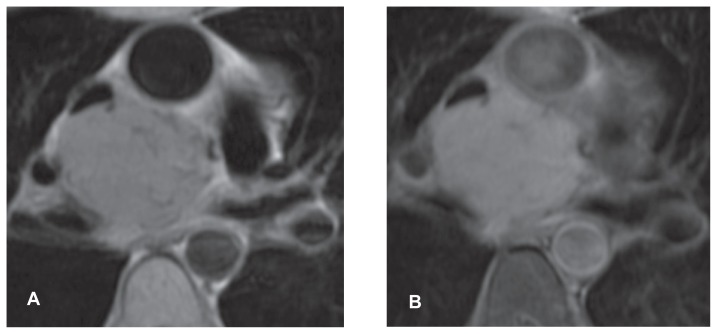
**A, B** T1 weighted double inversion recovery pre and post gadolinium images (image **B** additionally acquired with fat saturation). The second image was acquired approximately 3 minutes after contrast administration. Increase in signal is seen compared to the pre-contrast image **A**) but is much less intense than that seen during the dynamic acquisition (Fig. 2).

Post-contrast T1 weighted imaging confirmed a persistent increase in signal intensity within the mass (Fig. 3). Phase velocity mapping also appeared to demonstrate flow within the areas of tubular signal void described (not shown).

## Discussion

Castleman disease is a rare, generally benign, lymphoproliferative disease first described in 1958 (1). Two basic histological types have been described. The hyaline vascular type is more common, occurring in up to 90% of cases. The remaining cases are of the plasma cell variety which has a greater tendency to be associated with systemic symptoms, multifocal tumour deposits and a more aggressive clinical course (2).

The majority of unicentric cases occur within the mediastinum (3). Thoracic disease generally occurs within the mediastinum or at the pulmonary hila although cases have also been reported in pleural fissures and intercostal spaces (4,5).

Our patient was also unfortunate in suffering from pemphigus vulgaris and bronchiolitis obliterans. The association between paraneoplastic pemphigus and Castleman disease has been reported in animal models (6). Patients with paraneoplastic pemphigus may have auto-antibodies directed against their own epithelial proteins (7,8). The link with obliterative lung disease has been proposed as a proportion of patients will have antibodies directed against their respiratory epithelial cells (9). In the largest published series of patients with both paraneoplastic pemphigus and Castleman disease, 26 out of 28 subjects developed obliterative bronchiolitis with a fatal outcome in 22 cases (10). Our patient similarly developed this condition and eventually required a double lung transplant.

**Figure 4:**
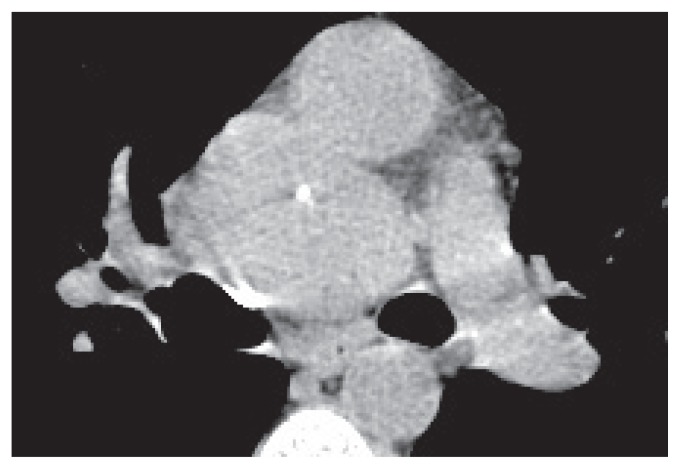
Unenhanced axial CT image demonstrating the Castleman nodal mass abutting the left main pulmonary artery and ascending aorta. A central spot of calcification is present.

**Figure 5:**
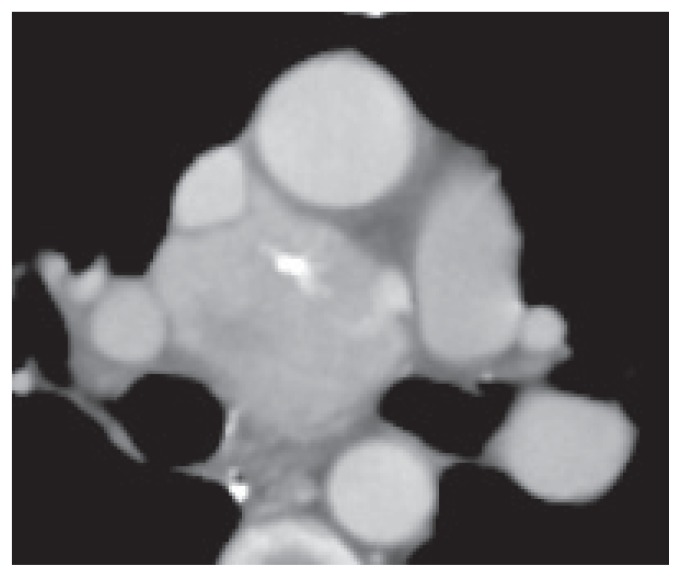
Post contrast axial CT image of the Castleman mass. Enhancement is fairly uniform and again a calcified focus is seen. Even though this is an arterial phase acquisition the intense vascularity of the mass is less well appreciated than on the dynamic MRI sequence (Fig. 2 images/movie). Note that this image was acquired 8 years after that shown in Figure 4, demonstrating the lack of disease progression in this patient.

Most reports feature descriptions of the computed tomographic appearances of the tumor. Unenhanced lesions are of soft tissue attenuation and may contain calcium, perhaps as an indication of their chronicity (11)—features seen also in our patient at the time of diagnosis (Fig. 4). Calcifications may vary in size and appearance being punctate, coarse, linear or arborising (12). On enhanced CT, Castleman disease has been reported to demonstrate significant, uniform contrast enhancement (Fig. 5) although the relationship of image acquisition to timing of contrast administration is rarely discussed (12,13). Conversely it has been suggested that even in the hyaline vascular type, enhancement may not be dramatic (14). In some series, central non-enhancing “scars” have been described (15). Several authors describe a less common mode of enhancement progressing from the periphery to the center of the lesion (16,17)—this was not apparent on dynamic imaging in our case (see Fig. 2 movie).

The MR features of Castleman disease in many ways mirror the appearances seen at CT (18,19) and are rather non-specific. In most cases the mass is described as being of similar signal intensity to skeletal muscle on T1 weighted imaging, with increased signal on T2 weighted images and following gadolinium enhancement (20). A number of these reports comment on the presence of linear hypointense regions within the mass which fail to enhance—a finding also seen in our patient. It is uncertain what this appearance is due to. Calcification seems unlikely as there was no evidence of “blooming” on the gradient echo images. Another possibility is that these represent areas of fibrosis (18) and this is confirmed in the series from Zhou et al. where pathological examination demonstrated swathes of fibrous tissue corresponding to low signal areas (16).

There is little mention in the literature of the optimal temporal relationship between administration of contrast and image acquisition in order to maximize visibility of the nodal mass in Castleman disease. Clearly, in most cases, post contrast imaging is occurring at a time point 50–60 seconds or more after injection. Since this is during the venous or later phase of contrast transit through the lesion, early washout may help to explain the variable enhancement on CT reported by some investigators (14).

Conventional MR imaging of the thorax for the purpose of lesion characterization generally involves variably-weighted spin-echo sequences before and after the administration of intravenous gadolinium contrast. Newer sequences for thoracic imaging have been developed primarily to aid imaging of the beating heart. Myocardial perfusion is one area which has received particular attention. In order to perform time-resolved imaging of a first pass bolus of contrast agent, it is necessary to acquire multiple angiographic data sets quickly and sequentially over a moderate-sized field of view. The necessary trade-offs to permit this include decreased spatial resolution due to thicker slices and a low resolution matrix resulting in a noisier image.

Nevertheless, the inherently high contrast resolution of the technique compensates sufficiently for other deficiencies if the structure under examination is of reasonable size. Additionally, the perfusion sequence we employed maximizes contrast by applying an initial saturation pre-pulse to the volume acquired which nulls signal from static tissue. Motion-related blurring is not an issue as this is a gated acquisition, unlike most conventional MRA pulse sequences.

In the case presented, the dynamic perfusion sequence was employed simply out of interest as we give gadolinium for T1 weighted imaging as part of a standard tumour protocol. Since we had not anticipated the diagnosis in advance, we were initially surprised to see the intense and transient enhancement of the lesion. Indeed, without the benefit of other sequences it would have been easy to believe that we were looking at an aneurysmal abnormality relating to one of the vascular structures within the mediastinum.

The differential diagnosis of mediastinal masses is wide and includes both benign and malignant lesions of the bronchi, pericardium, neural tissue, nodal tissue, thyroid and thymus. Pathology of the aorta, pulmonary arteries and even the coronary arteries may also on occasion give rise to mediastinal abnormalities. Castleman nodal tissue can be so vascular that the dense enhancement seen with an angiographic sequence may misleadingly suggest a primary vascular abnormality. However, with a solid appearance on standard imaging sequences, the profound early enhancement on a dynamic sequence is a strong pointer towards the final diagnosis. One other report in the literature describes a similar technique employing time-resolved magnetic resonance angiography for assessment of unusual liver lesions; in that report the pronounced arterial phase enhancement seen again allowed the correct diagnosis of Castleman disease to be made (21).

Although a wide differential diagnosis was considered in our case, it was felt that the combination of previous documented histology and typical imaging findings made any other diagnosis unlikely. The patient’s attending physician was in agreement and the mass has simply been monitored (without biopsy) in subsequent years (Fig. 5).

We suggest that if Castleman disease is being entertained as a clinical diagnosis, a dynamic sequence may reveal the inherent vascularity of the lesion to a far greater extent than regular post-contrast spin echo imaging. To our knowledge, this is the first time that a gated sequence designed for myocardial perfusion has been used in this way.

## Figures and Tables

**Table 1:** Technical parameters pertaining to the dynamic perfusion sequence.

Field of view	38 cm coronal acquisition
Number of slices	5
Slice thickness	8 mm, no gap
Matrix	128 × 128
Parameters	TR 6.6, TE 1.4
Sequence	Fast gradient echo with echo-train read-out (FGRET)
Number of phases	40
Gadolinum dose	10 ml (0.2 mmol/ml)
Injection rate	5 ml/sec
Saline chaser	25 ml at 5 ml/sec
